# Death of Charlotte Bronte

**Published:** 1857-11

**Authors:** Walter Channing


### *Death of Charlotte Brontë.*

The death of Charlotte Brontë is the saddest fact in a life whose key-note was sorrow, and whose melancholy music filled the very atmosphere in which she lived, and moved, and had her being. She may almost be said to have been baptized in the dark waters of death. Her mother died when she was about five years of age, and, in quick succession, four sisters and her only brother.

It was not a common family, that of Charlotte Brontë. Two of her sisters died young, but lived long enough to indicate that they would have left their mark on their times. The two elder sisters gave the same evidence of their power in written works. Her brother had large intellectual endowment and culture, but worse than wasted all that might have greatly distinguished him. We do not design in this notice of one whose life has been so admirably written by Mrs. Gaskill, and which all readers have read, to review this work. And yet it may not be out of place to say that it is a record of a remarkable person, who in the midst and pressure of severe trial, never failed in duty to herself, and to all to whose well-being she could in any way contribute. She was small, delicate in person—apparently incapable of effort. Yet she meets or makes occasion for intellectual, moral and physical action, which in its detail astonishes us by its rarity, and still more by its success. She writes with startling strength—brings before you men and women, her own creations, and reveals what is in them, both in their word or work, in langnage and act which leaves little ground for question. She goes to a foreign country, of different language from her own —goes alone, by the guidance of the same instinct which always accompanies a true object, and accomplishes all she attempts. She writes, and while her manuscripts are gathering dust on the publisher's shelves, she writes on, nothing daunted, and at length comes forth as an author, and declares, anonymously, her gigantic power. "Who wrote Jane Eyre?" is the question. "Not a man," says one, "for a man would not"— "Not a woman," says another, "for a woman could not."

Pardon us, that we have for a moment deviated from our purpose— to speak of the death of Charlotte Brontë. We could not but say a word of a life so sad as was hers, and for the reason that in an event which was to her an unmixed felicity, she found death. Sadly, in deep sadness, do we ask, was it not a fitting coronation of such a genius, and such a life?

Charlotte Brontë married late in life. Her father opposed her marriage, and the daughter could not marry the man she so deeply loved, as her marriage must separate her from her father, now more than eighty years of age, and with no living creature of his house, but her, left.— At last, her father's consent is given and she is married This was an event in Haworth. Every body came to the wedding. Charlotte had been the friend of all the poor. She would traverse, in snow and rain, the wild moors of her home, to carry something for the sick child or parent, or to do something for them. Every body knew her, and every loved her. Says Mrs. Gaskill, "many old and humble friends were there, seeing her look like a snow-drop." Her bridal dress, after a few months, became her shroud.

She became pregnant, and soon after experienced the ordinary symptoms of that state, but which rapidly became morbidly severe. Nausea, vomiting and faintness; and fainting, at first frequent, became, at length, constant. The sight of food was sufficient to produce them all in most distressing forms. Said one, "a wren would have starved on what she ate during those last six weeks." A physician was called.— "He came, and assigned a natural cause for her miserable indisposition; a little patience, and all would go right."

From the record, nothing more seems to have been said or done in this case. We copy the following from Mrs. Gaskill, because of its professional interest, and as showing something of the sufferer's state in the last moments of her life.

"Long days and longer nights went by; still the same relentless nausea and faintness, and still borne on in patient trust. About the third week in March, (it was early in the new year, 1855, that the symptoms first appeared,) there was a change; a low, wandering delirium came on; and in it she begged constantly for food, and even for stimulants. She swallowed eagerly now; but it was too late. Wakening, for an instant, from this stupor of intelligence, she saw her husband's woe-worn face, and caught the sound of some murmured words of prayer that God would spare her. 'Oh!' she whispered forth, 'I am not going to die, am I? He will not separate us, we have been so happy.'"

She died Saturday morning, March 31st.

It is of the professional relations of our subject—the treatment of the signs of pregnancy when morbidly aggravated, that we would now speak. Was the *cause*, the *motive cause* of those symptoms which produced death in Charlotte Brontë, removed? This question is of great interest. Nearly half a century ago, it was our privilege to attend the midwifery lectures of Dr. John Haighton, in London; and a better lecturer than Haighton, is not in our memory. He discussed this question of removing the *cause* of those symptoms, and showed conclusively that in cases in which other means had failed, and the worst consequences were to be looked for, it was the duty of the physician to remove the *cause*, viz: *to remove the fœtus from the womb.* Haighton related his experience, and dwelt on the opposition he had met with in consultations, to such measures as he knew could alone save life. More recently we have spoken with eminent men abroad, on this subject, and have met with objections to the practice; or, when it has been allowed to be proper, it has been after so much evil has been done that there has hardly been any reason to look for success from it.

We have felt it our duty to resort to the measure under consideration, and in every case recovery has been rapid and complete. We have known death to happen when the measure has been rejected by patient or friends, and where all other means have been faithfully used. In one case it was clear that death must occur, if things remained as they were, but in which the mother of the patient would not consent to the measure, unless the physicians who advised it would in the first place guarantee its success. The attending physician would not do this; and soon after our consultation we heard of the patient's death.

In another instance, the lady lived in a distant State. She was a clergyman's wife, and of the Church of England. She was reduced by nausea and vomiting to excessive weakness, and absolutely could keep nothing on her stomach. It was between the second and third month of pregnancy. The fœtus was removed, and, in twenty-four hours after, we found her heartily eating solid food, and she was soon well. The operation was performed on the same patient a second time under the same circumstances, and with the same result. Let it be remembered that this practice was not attempted until full trial had been made of the most approved methods of treatment, and after the best evidence that the disease was rapidly increasing. In another lady it was not until convulsive movements had occurred in the universal exhaustion, that the measure was adopted. This patient recovered, and this was a second trial of it in the same patient.

We dwell on these cases, because a grave moral question is involved in our subject; and to say that it is only in those cases in which life is clearly in jeopardy, that any physician who deserves the name, would for a moment entertain the question we are considering. It is then as a *remedy;* and only to be used under what we believe are really desperate circumstances.

Whether the cause was removed in Charlotte Brontë's case, or whether she died of pregnancy, we know not. We know not what was the limit of that "little patience, when all would go right." But as the disease continued unrelieved till death, may it not be asked if the *cause* of that disease did not remain undisturbed till it became the cause of death?—The question is put, because in no like case which has come under our care, however unpromising, has death occurred after the removal of the contents of the womb.

The Rectory at Haworth is now desolate. Its venerable head, in his extreme age, stands erect and alone, literally in the midst of the graves of all his house; and before him, in his church, is the simple tablet on which are recorded the names, the ages and the death, of his wife and of all his children. WALTER CHANNING.

[*Boston Med. & Surg. Journal.*

